# Sequencing of the needle transcriptome from Norway spruce (*Picea abies* Karst L.) reveals lower substitution rates, but similar selective constraints in gymnosperms and angiosperms

**DOI:** 10.1186/1471-2164-13-589

**Published:** 2012-11-02

**Authors:** Jun Chen, Severin Uebbing, Niclas Gyllenstrand, Ulf Lagercrantz, Martin Lascoux, Thomas Källman

**Affiliations:** 1 Department of Ecology and Genetics, EBC, Uppsala University, 752 36 Uppsala, Sweden; 2 Department of Plant Biology and Forest Genetics, Swedish University of Agricultural Sciences, Uppsala, P.O. Box 7080, SE-750 07 Uppsala, Sweden; 3 Laboratory of Evolutionary Genomics, CAS-MPG Partner Institute for Computational Biology, Chinese Academy of Sciences, Shanghai, China

## Abstract

**Background:**

A detailed knowledge about spatial and temporal gene expression is important for understanding both the function of genes and their evolution. For the vast majority of species, transcriptomes are still largely uncharacterized and even in those where substantial information is available it is often in the form of partially sequenced transcriptomes. With the development of next generation sequencing, a single experiment can now simultaneously identify the transcribed part of a species genome and estimate levels of gene expression.

**Results:**

mRNA from actively growing needles of Norway spruce (*Picea abies*) was sequenced using next generation sequencing technology. In total, close to 70 million fragments with a length of 76 bp were sequenced resulting in 5 Gbp of raw data. A *de novo* assembly of these reads, together with publicly available expressed sequence tag (EST) data from Norway spruce, was used to create a reference transcriptome. Of the 38,419 PUTs (putative unique transcripts) longer than 150 bp in this reference assembly, 83.5% show similarity to ESTs from other spruce species and of the remaining PUTs, 3,704 show similarity to protein sequences from other plant species, leaving 4,167 PUTs with limited similarity to currently available plant proteins. By predicting coding frames and comparing not only the Norway spruce PUTs, but also PUTs from the close relatives *Picea glauca* and *Picea sitchensis* to both *Pinus taeda* and *Taxus mairei*, we obtained estimates of synonymous and non-synonymous divergence among conifer species. In addition, we detected close to 15,000 SNPs of high quality and estimated gene expression differences between samples collected under dark and light conditions.

**Conclusions:**

Our study yielded a large number of single nucleotide polymorphisms as well as estimates of gene expression on transcriptome scale. In agreement with a recent study we find that the synonymous substitution rate per year (0.6 × 10^−09^ and 1.1 × 10^−09^) is an order of magnitude smaller than values reported for angiosperm herbs. However, if one takes generation time into account, most of this difference disappears. The estimates of the dN/dS ratio (non-synonymous over synonymous divergence) reported here are in general much lower than 1 and only a few genes showed a ratio larger than 1.

## Background

A detailed characterization of the transcriptome is not only a prerequisite for genome annotation, but is also crucial to relate gene expression and function to phenotypic variation, to estimate evolutionary rates or to identify genes under selection [1-5]. While the first large-scale EST (Expressed Sequence Tag) projects based on Sanger sequencing had already revealed that transcriptomes are highly diverse and complex, this has over the last decade been corroborated on an unprecedented scale by massive parallel sequencing (MPS) [6-9].

Until recently microarrays, based on either cDNA, or in the case of model organisms, oligonucleotides, were the main tool to assess global patterns of gene expression. These microarray studies provided a first characterization of temporal and spatial gene expression patterns in many different organisms, but they also had obvious limitations since they were dependent on already available sequence information from the organism of interest. Furthermore, at least with cDNA arrays, most transcripts were only represented by the most common splice variant making the gene expression estimates unspecific [10]. The emergence of MPS techniques has profoundly transformed the landscape of both genome and transcriptome sequencing [11]. In the case of transcriptome sequencing it has become possible to obtain both sequence data and estimates of gene expression levels in a single experiment [12]. MPS also allowed for very deep sequencing of transcriptomes revealing in principle all expressed parts of the genome. In mouse (*Mus musculus*), for example, despite a fully sequenced genome and millions of EST sequences in public databases, MPS of expressed genes from three different tissues revealed several thousand new features of known genes and close to 600 novel gene transcripts [6]. Similar results, with a very large number of previously unknown expressed parts of the genome, were recently obtained in *Drosophila melanogaster*[13]. In plants, MPS has been used to investigate gene expression and alternative splicing in model organisms. As in animals, large parts of the genome are transcribed (although the majority of transcripts can be assigned to previously identified expressed parts of the genome) and around 30-40% of the genes have more than one expressed splice variant [8,9]. In addition, transcriptome sequencing has been employed in a large array of plant species to create basic genetic resources and to study gene expression dynamics (for example see: [14-20]).

Most efforts have so far focused on angiosperms, less attention being paid to gymnosperms in spite of the availability of several very large EST libraries in the spruce (*Picea*) and pine (*Pinus*) genus. By April 2012 there were 313,110 and 186,637 EST sequences deposited at NCBI from *Picea glauca* and *P. sitchensis*, respectively. Efforts in the European *Picea abies* (Norway spruce) have been less intensive and only some 14,000 ESTs had been deposited at NCBI by April 2012. Based on sequence similarity, the data obtained in the North American species have been collapsed into 22,472 putative unique transcripts (PUTs, unigenes) in *P. glauca* and 19,828 unigenes in *P. sitchensis* (see Unigene assembly on NCBI). In addition, more focused experimental and bioinformatic efforts have been made to obtain full-length cDNAs (FL-cDNA) in both *P. sitchensis* and *P. glauca* and have so far lead to the identification of 27,720 (23,589 annotated as FL-cDNA) unique transcribed genes in *P. glauca* and around 8,000 validated FL-cDNA have been characterized in *P. sitchensis*[21,22]. These data sets have been very useful for creating microarray assays, detecting polymorphic markers to construct genetic maps and to estimate evolutionary rates [23-26]. Gymnosperms separated from angiosperms some 300 million years ago [27] and they differ in many important aspects. For example, in conifers, the overall recombination seems to be lower than in angiosperms and limited to genes [28,29] and, in contrast to angiosperms where genome duplication has been pervasive, genome duplication is rare in conifers [30]. The generally very large size of conifer genomes is rather a result from massive invasion of transposable elements early in the history of the group [31]. One could therefore expect different mode and tempo of evolution of the transcriptome in these two groups of plants. The gymnosperm group does, compared to the angiosperm group, contain fewer species, but many gymnosperm species have very large distribution ranges and are of great ecological and economical importance [32]. The largest taxon within gymnosperms is the coniferophyta that harbors around 600 species, including many tree species such as pines, spruces and yews [27]. Within these three groups the phylogenetic relationships are not fully known since molecular divergence tends to be very low. For example, within the spruce genera morphological and molecular markers often give conflicting results and multi-locus nuclear DNA data indicates that incomplete lineage sorting is a major challenge for inferring species trees [33]. The spruce lineage separated from the pine lineage around 120-160 millions years ago whereas the Taxus lineage is more distantly related and are believed to have a common ancestor to the pine and spruce lineages between 240 and 300 million years ago [27,34].

In this study we present results from deep sequencing of the *P. abies* needle transcriptome. The data was used to create a reference transcriptome, containing a extensive set of genes expressed in actively growing spruce needles. Combined with available EST from publicly available databases, the size of the characterized part of the transcriptome in *P. abies* now reaches the same order of magnitude as data available from North American spruce species, with thousands of putative full-length genes. We used these data to address the following questions: First, to what extent can this type of short sequence data be assembled *de novo* to full length transcripts? Second, can the short reads be used to identify polymorphic sites? Third, by analyzing mRNA samples from two time points; during day (in the light) and during night (in the dark) can we detect differentially expressed transcripts and if so does the inferred pattern differ compared to angiosperms? Fourth, since recent studies of divergence between conifer species have suggested that there might be major differences in evolutionary rates between conifers and angiosperms [26] we used the assembly together with data from *P. glauca*, *P. sitchensis*, *Pinus taeda* and *Taxus mairei* to evaluate this statement with a larger and more diverse data set. By comparing not only our, but also two other spruce transcriptome sequences created by traditional sequencing methods, to both *Pinus taeda* and *Taxus mairei* we could minimize biases that might exist in any single assembly. Furthermore, since inferring pattern of divergence between species can be biased when closely related species (*e.g.* incomplete lineage sorting) or highly divergent species (saturation on synonymous sites) are used, the use of species that diverged at two different time points should reduce these problems.

## Results

RNA extracted from needles of a single adult *P. abies* tree growing outside Uppsala, Sweden (59°51’29”N, 17°48’38”E) was used to produce 6 different adapter-ligated cDNA libraries. In total, close to 70 million 76 bp long fragments were sequenced from both ends and close to 50% of the reads originated from the samples collected in the dark (Table 1). Based on overlap between pair-end reads, the obtained library sizes were smaller than the expected 150-300 bp and around 50% of the reads contained detectable traces of adaptor sequence, which means that the fragment size was smaller than the read length of 76 bp. Thus, reads were filtered, not only based on quality, but also on presence of adaptor sequence, reducing the data set to 33,185,901 fragments that was used in *de novo* assembly (∼15 million where paired end sequences were retained and ∼18 million when only one of the paired reads was retained). For mapping purposes we created a second data set where reads containing adaptor sequence were trimmed rather than removed and retained if they were longer than 30 bp after trimming. This created a data set of 55,417,522 fragments (almost 36 millions sequenced at both ends and 19.5 millions from only one end) of length between 30 and 76 bp (Table 1).

**Table 1:** Summary of the collected sequence data

		**Used in *****de novo *****assembly**		**Used in mapping**	
**RNA source**	**Raw PE reads**	**PE reads**	**SE reads**	**PE reads**	**SE reads**
Light extraction 1	28,073,980	5,668,652	3,229,442	14,751,144	3,738,405
Light extraction 2	20,442,438	4,589,450	2,930,651	9,693,554	3,077,721
Light extraction 3	23,554,246	5,183,788	2,929,447	12,575,038	3,217,660
Dark extraction 1	23,885,794	5,004,782	3,054,332	12,080,884	3,359,437
Dark extraction 2	22,157,136	4,929,488	2,929,710	11,349,636	3,084,920
Dark extraction 3	21,643,358	4,981,140	2,933,669	11,307,910	3,060,296
**Total**	**139,756,952**	**30,357,300**	**18,007,251**	**71,758,166**	**19,538,439**

Collected number of sequences from the 6 different sequence libraries. The number of reads used for assembly and mapping divided as pair-end (PE) and single-end (SE) reads retained after cleaning and filtering as described in Material and methods.

### *de novo* assembly

**Figure 1:**
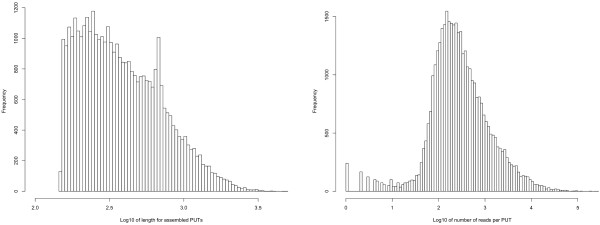
**Distribution of length and coverage of PUTs.** Histogram of length (left) and number of reads per PUT (right). To aid visualization both PUT length and read count values were log10 transformed. Note that all PUTs shorter than 150bp were excluded from the histograms.

Two types of short read *de novo* assemblers were used. First, both pair-end and single-end reads were assembled using Velvet [35] and second, all reads were treated as single-end reads and assembled using ABySS [36]. The resulting contigs from both assemblers were merged with 8,715 PUTs (putative unique transcripts) assembled from ESTs at plantgdb.org [37] to create a more comprehensive set of PUTs from *P. abies*. The final assembly contained 59,556 PUTs with a N50 and a N90 size of 551 bp and 156 bp, respectively (N50 and N90 are the length of the shortest contigs in a set of contigs sorted in a descending order that includes 50% and 90% of the total assembly length). In all downstream analyses we excluded PUTs shorter than 151 bp, leaving 38,419 PUTs with a mean length of 471.5 bp. The total assembly length of PUTs longer than 150 bp was just over 18 Mbp and the mean sequence depth of these PUTs as measured by RPKM (reads per kilobase of transcripts per million of mapped reads) was 281 (Figure 1 and Additional file 1).

55% of the assembled *P. abies* PUTs showed similarity (blastx E-value  < 1 × 10^−10^) to plant proteins when translated to protein sequences. Of the 38,419 PUTs 32,082 show a significant hit against either *P. glauca* or *P. sitchensis* and 23,450 of these were similar over more than 90% of the sequence length of the *P. abies* PUT. Of the 882 transcripts longer than 1,500 bp only 57 PUTs did not show any similarity to Arabidopsis or other plant proteins. Taken together this left only 4,167 *P. abies* PUTs with no or limited similarity to any plant protein sequence or any EST sequence from other spruce species. For complete annotation of PUTs longer than 150 bp against *A. thaliana*, *P. glauca*, *P. sitchensis*, plant proteins, PFAM databases and repeat databases see Additional file 1.

**Figure 2:**
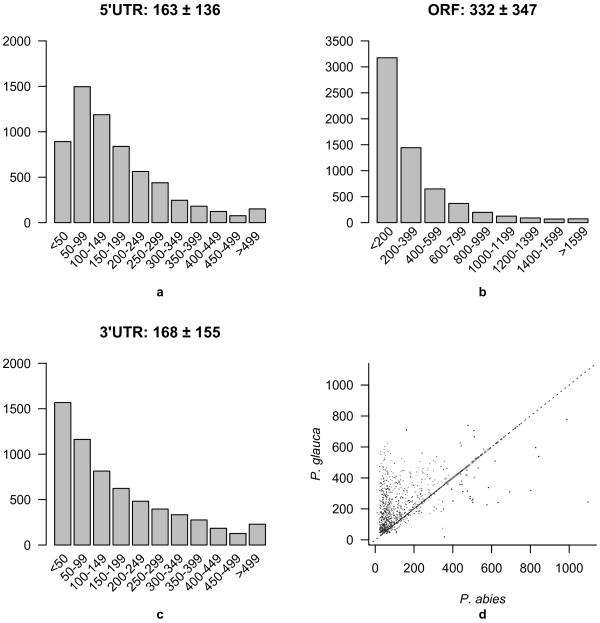
**Length distributions of different mRNA features in the *P. abies *PUTs.a**) 5’-UTR (untranslated region). **b**) ORFs (open reading frame). **c**) 3’-UTR. **d**) Comparison of putative full-length ORFs in *P. abies* and *P. glauca*.

**Figure 3:**
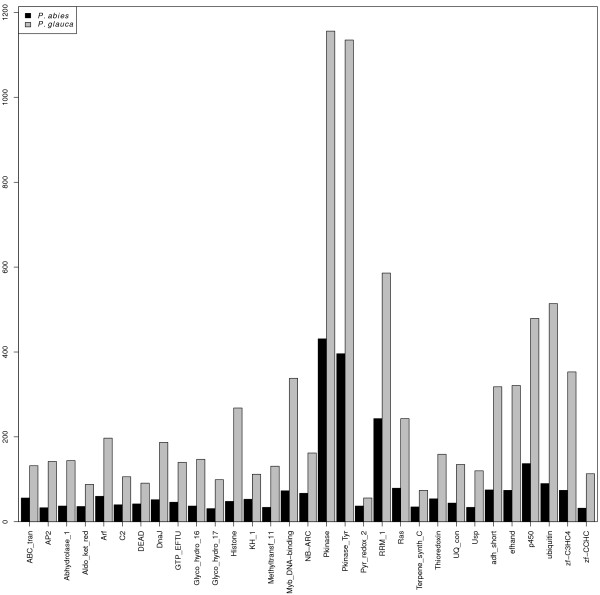
**Histogram showing domain predictions from ORFs in *P. abies *and *P. glauca*.** The proteins domain names are from the PFAM-A database.

Potential reading frames were identified from all PUTs longer than 150 nucleotides. This led to the identification of 6,194 PUTs that have properties suggesting that they are full-length transcripts including both a start and stop codon and have an open reading frame (ORF) of at least 10 amino acids (Additional file 1). Comparison of the length distribution of both untranslated regions (UTRs) and the ORF length to the validated full-length data set from both *P. sitchensis*[21] and *P. glauca*[22] suggests that a fraction of our putative full-length PUTs includes full-length transcripts. However, the length distributions of both the 5’-UTRs, 3’-UTRs and ORFs were enriched for short sequences compared to what was reported from the *P. sitchensis* and *P. glauca* (Figure 2 compared to Figure three in [21]). A direct comparison between a set of potentially orthologous sequences in *P. abies* and *P. glauca* shows that there is a large number of PUTs that likely are full-length as they are equally long in both species, but it is also clear from this comparison that neither of these two data sets are solely composed of full-length transcripts as many transcripts annotated as full-length in either *P. glauca* or *P. abies* appear to be truncated when compared to each other (Figure 2d). Approximately one fifth of the 6,194 full length *P. abies* transcripts where orthologous sequences were found in *P. glauca* displayed less than 5% difference in sequence length and hence likely represents full length transcripts. All PUTs containing coding frames were also annotated by comparing them to the PFAM-A domains. A comparison of the number of different detectable domains in the *P. abies* transcriptome to the *P. glauca* transcriptome is consistent with shorter length of assembled *P. abies* transcriptome. We detected fewer domains of all types in the *P. abies* assembly, but the distributions from the two species are indeed very similar (Figure 3).

### Identification of variable sites

By mapping short reads to the *P. abies* PUT assembly we extracted potential SNPs using samtools and the mpileup function on the mapped reads. The number of SNPs identified was, as expected, largely dependent on the criteria used in the algorithm and as the data is from non-normalized RNA the commonly used settings for genome sequencing will likely not give satisfactory results. With very stringent criteria, including a cut-off for the observed allele frequency of the minor allele at 25% (note that as all the data is from one individual we expect segregating alleles to be found at frequency 0.5 unless there are allele specific gene expression), read direction for alternate alleles (DP4) and coverage 9,394 SNPs were found. Less stringent criteria obviously led to a higher number of identified SNPs and if we only filter on quality 20,781 potential SNPs were identified. From published *P. abies* population genetic data set the observed transition to transversion ratio is around 1.3, which is very similar to the ratio observed with the criteria that includes both quality and observed minor allele frequency above 0.25 (Table 2).

**Table 2:** Putative single nucleotide polymorphisms at different quality criteria

**Filter**	**SNP**	**Transitions**	**Transversions**	**Ratio**
Sanger Qual > = 60	20,781	11,389	9,392	1.21
Sanger Qual > = 60 and AF [0.25, 0.75]	14,745	8,372	6,372	1.31
Sanger Qual > = 60 and AF [0.25, 0.75]	9,394	5,622	3,770	1.49
and Depth > = 20, DP4> = 10				

The effect of using different quality criteria for identifying SNPs. Sanger Qual = Sanger sequence quality, AF = Allele frequency, Depth = sequence depth at the base DP4 = Depth of the two variants according to the read direction. For details see the samtools specifications.

### Comparative molecular evolution in conifers

**Figure 4:**
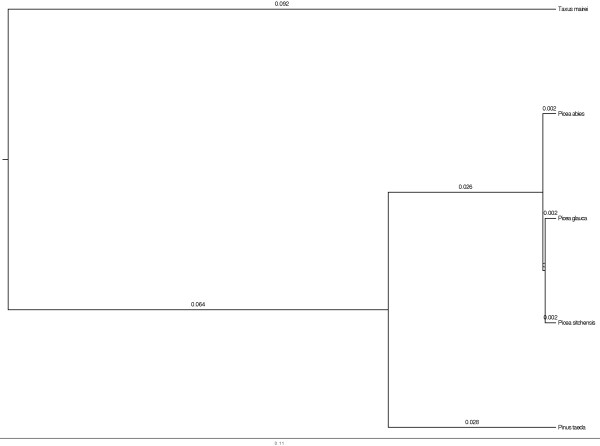
**Phylogenetic tree showing the relationship between the five conifer species included in the study.** Branch lengths represent inferred genetic distance.

**Figure 5:**
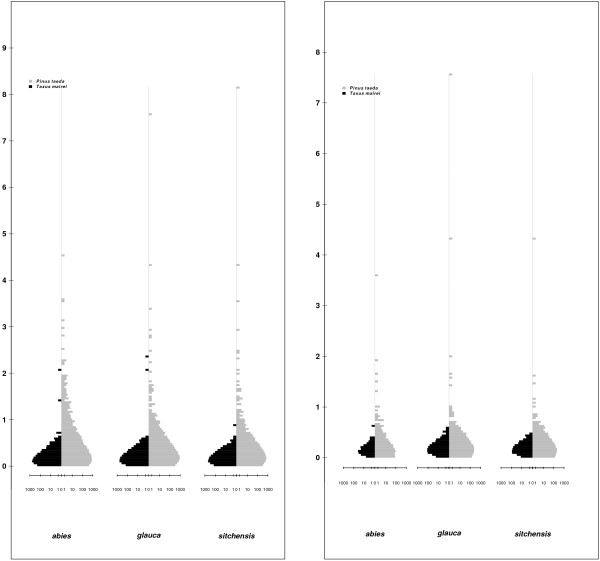
**Histograms showing the distribution of dN/dS values from pairwise comparisons of species.** The left plot shows pattern for all potential orthologous sequences in the data set whereas the right plot shows the pattern when restricting the data sets to only putative full-length ORFs.

Previous genetic studies from both nuclear and organellar markers within the *Picea* group have revealed a fairly shallow phylogeny with partly contrasting results between different marker types [33,38-40]. All these studies have been restricted to either small data sets using at most 10 gene fragments or only organelle markers. Here we combine EST data from three spruce species (*P. abies*, *P. glauca* and *P. sitchensis*), a yew (*Taxus mairei*) and a pine (*Pinus taeda*) to estimate phylogenetic trees and study the rate of molecular evolution in conifers. In all five species we identified putative coding frames and aligned orthologous sequences from all species. This resulted in a data set of 5,246 aligned ORFs between the three *Picea* species. This data set was reduced to 1,404 when adding pine and further reduced to 244 when adding yew. Phylogenetic inference on this latter data set confirms the very close relationship between all three spruce species and we cannot with any confidence resolve their phylogeny (Figure 4). Due to this lack of resolution and the large reduction in data sets as more species were added, we restricted the analysis of synonymous and nonsynonymous divergence to pairwise comparisons between one of the three spruce species, on the one hand, and either the pine or the yew, on the other hand. This created 6 pairwise data sets with an average 4,737 ORFs aligned in each comparison (*P. abies* - *P. taeda*: 6,977, *P. abies - T. mairei*: 2,932, *P. glauca* - *P. taeda*: 7,183, *P. glauca* - *T. mairei*: 3,110, *P. sitchensis* - *P. taeda*: 5,773, *P. sitchensis* - *T. mairei*: 2,447). Mean synonymous divergences between the three spruce species and pine and yew were 0.175 (*P. abies*: 0.172 [95% confidence interval, CI = 0.1701, 0.1736], *P. glauca*: 0.177 [CI = 0.1752, 0.1783], *P. sitchensis*: 0.176 [CI = 0.1743, 0.1779]) and 0.598 (*P. abies*: 0.577 [CI = 0.5711, 0.5837], *P. glauca*: 0.605 [CI = 0.5979, 0.6120], *P. sitchensis*: 0.611 [CI = 0.6028, 0.6190]), respectively. Assuming a divergence time between pine and spruce of 160 to 120 million years [26,34] and a divergence between spruce and yew of 300 to 240 million years [27] these values correspond to a yearly substitution rate of 0.55 × 10^−09^ to 0.73 × 10^−09^ and 1 × 10^−09^ to 1.24 × 10^−09^, respectively. The ratio of non-synonymous to synonymous divergence between the spruce species and pine and yew show very few genes with ratios larger than one, irrespective if the analysis were performed on only full-length ORFs or on all identified ORFs (Figure 5).

### Differentially expressed genes

**Figure 6:**
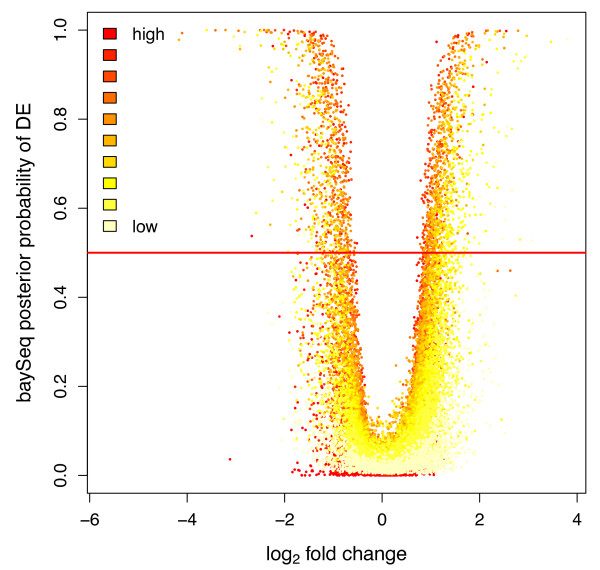
**Volcano plot of gene expression pattern.** The y-axis represent the posterior probability of differential expression and the fold change in log2 on the x-axis. Dots are colored to represent estimated expression level. The red line indicate a cut-off value of 0.5 for the posterior probability of differential expression.

Since sequence data were collected from three technical replicates sampled in the dark during night and three replicates sampled during daytime in normal daylight conditions, we set out to detect differentially expressed genes. The correlations of estimated levels of gene expression based on just counting the number of pair-end hits against any given reference gene were in general very high especially for the dark samples (data not shown). 2,076 transcripts were detected as significantly differentially expressed between the two sampling points using BaySeq and a cut-off posterior probability at 0.5, but among these transcripts only few showed a large fold change (Figure 6, Additional file 1). We did not observe any significant correlation between either expression level or differential expression and selective constraint as measured by dN/dS.

## Discussion

In spite of the remaining challenges associated to the assembly of short reads to full-length transcripts [41] RNA-seq has over the last years evolved into the de-facto standard for transcriptome analysis even in non-model species [11,42]. In the present study we evaluated the quality of our assembly using a set of different criteria largely relying on comparisons to other plant species. First, we compared basic summary statistics to data from other conifer species, where the transcriptome was obtained through Sanger sequencing. Second, we further characterized the assembly by assessing properties like transcriptome annotation and similarity at the protein level to available plant protein sequences. *P. glauca*, which has one of the best characterized transcriptome among conifer species, has a transcriptome assembly length of 30.15 Mbp, i.e. 12 Mbp longer than the the *P. abies* assembly presented here. In addition, the length of individual PUTs is on average shorter in the *P. abies* assembly than in both *P. glauca* and *P. taeda* (Figure 1, Figure 2, http://www.plantgdb.org). This indicates that the *P. abies* transcriptome assembly only comprises a fraction of the total transcriptome and that a large proportion of the assembled PUTs corresponds to partial transcripts. There are a number of possible reasons for this difference in assembly length despite of the large amount of short reads collected. First, the assembly of short reads from transcriptome sequence is still far from optimal [41], something that likely is elevated in this data set as the mean fragment length in the sequence library was shorter than the pair-end distance. Second, non-normalized mRNA was used, which means that the read depth of lowly expressed genes is likely insufficient for assembly. Third, the short read libraries used here obviously will capture only a restricted part of the total transcriptome as they are based on a single tissue and only two sampling time points. All this is in contrast to the other mentioned conifer species EST assemblies that are, at least partially, composed of normalized mRNA libraries and also include mRNA extracted from several tissues including root, which in general have divergent expression patterns [21,22,43,44].

In total over 34,000 transcripts showed significant hits when compared to other spruce species, either at the protein level to plant proteins or at the nucleotide level, leaving only 4,167 PUTs without any annotation. This set of PUTs with low or no similarity to other species could represent erroneously assembled PUTs, but since estimates from the *P. glauca* transcriptome suggest that as much as 11.6 Mbp of the transcriptome is still missing [22], they could also represent previously undiscovered spruce transcripts. In theory they could also be transcripts specific to *P. abies*, but this seems less likely as both this data and previous studies have shown that spruce species are very closely related and even share many polymorphic sites [33,39]. Still, genome size between different spruce species is variable (15.8 - 20.2 pg/1C [45]), which could imply significant differences in gene content between them. Finally, and perhaps most likely, they may represent assemblies containing mainly non-coding RNA and partial transcripts with mostly UTR sequence that, in general, show lower degree of conservation between species. In summary, even if the *P. abies* transcriptome contains a large fraction of partial transcripts and is far from complete, the assembly presented here is of a high enough quality and size to be informative on the general properties of the transcriptome.

In fact, ORF predictions identified 6,194 PUTs from *P. abies* with properties that suggest that they are full-length transcripts. A direct comparison of these PUTs towards full-length transcripts from *P. glauca* indicate that neither ours nor the *P. glauca* PUT assembly contains a comprehensive set of full-length transcripts and that despite millions of ESTs from spruce species there is still room for efforts aiming at capturing and sequencing full-length transcripts, such as oligo-capping, something that is of special importance in relation to upcoming genome annotations [46].

In many of the PUTs with high enough sequence coverage we could extract potentially variable sites, but since sequencing technologies are evolving at a rapid pace, the understanding of the error profiles for a given technique is still sparse making this process nontrivial and prone to false positives. This process is, in the present case, further complicated by the fact that the majority of tools developed to extract SNPs assumes that genome data is available and that sequence depth is even for the underlying genotypes, meaning that one expects around half the reads from each allele at heterozygous sites. In addition, many tools make use of uneven coverage as a tool to detect copy number variants or duplicated regions making standard methods to discover SNPs difficult to use as the uneven coverage and allele specific gene expression will cause deviations from these expectations. By merging population genetics sequence data sets from both unpublished and published projects in *P. abies*, where the data have been collected with Sanger sequencing [33,47] we compared the ratio of transitions to transversions to the ratio obtained here under different cut-offs. Adding both quality and coverage of the alternate alleles as criteria yielded almost identical transition to transversion ratio as the Sanger data, suggesting that this criteria might be suitable for this data set (Table 2). Hence, from a single individual study we have identified close to 15 thousand SNPs, but because all of these stem from only one individual a fraction of them will be either singletons or low frequency variants within the species. Even so these results suggests that mRNA sequencing can be of great use for the identification of population genetic markers. However, recent discussions have identified several issues in identification of variable sites from cDNA something that calls for caution and further validation before using SNPs from cDNA as a source for identification of genetic markers [48-50].

The observed mean synonymous divergence of 0.175 between spruce and pine obtained here, is similar to the 0.22 reported as a mean over a phylogenetic tree of four conifer species [3] and the 0.19 reported from a pairwise comparison of *P. sitchensis* and *P. taeda*[26]. In the comparisons to yew we obtained a mean silent divergence around 0.6. These divergence estimates translate into average synonymous substitution rates of 0.6 × 10^−09^ and 1.1 × 10^−09^, values that are also in line with previous estimates of 0.7 − 1.31 × 10^−09^[51]. As previously noted this yearly synonymous substitution rate is much lower than estimates of substitution rate from annual angiosperm species. Data from numerous studies have obtained similar patterns and there is now substantial evidence supporting a lower annual substitution rate in gymnosperms compared to many annual angiosperms [3,26,34,51]. Since lower synonymous substitution rates per year have also been observed in angiosperms trees/shrubs compared to herbs [52,53] one of the main causes seem simply to be that gymnosperms are trees with a long generation times and if substitution rates are calculated per generation rather than per year, the estimates between perennial gymnosperms presented here are similar to estimates from annual angiosperms. The mechanisms underlying the slower substitution rates in perennials than in annuals remain unclear [52,54] and we will not speculate on its causes here. Interestingly though, available data also suggest that polymorphism level is lower in trees, and in particular in conifers, than in herbs (Additional file 2). This apparent effect of generation time on nucleotide diversity could reflect the fact that organisms with long generation time and low substitution rate will require longer period of time to recover from past decline in effective population sizes. Also, for a given number of generations they will span much longer periods of time than annuals and thereby will be more likely to experience environmental changes causing variation in population size.

The ratio of non-synonymous to synonymous divergence at full length ORFs between spruce species and either pine or yew reported here, 0.236 (CI = 0.2272, 0.2432) and 0.167 (CI = 0.1614, 0.1717), respectively, are higher than estimates from previous analyses in conifer species [3], where branch specific estimates were used (with values of 0.12, 0.14 and 0.15 for the internal branch, the branch leading to *P. glauca* and the branch leading to *P. menziesii*, respectively), but lower than the pairwise estimate between *P. taeda* and *P. sitchensis* (0.314 (95% CI = 0.299, 0.329)) [26]. The deviation from the former study is likely due to the fact that they included only sequences present in all four conifer species used in their study thereby limiting severely the number of sequences (138) and likely enriching the data set for conserved sequences. The difference with the latter study is harder to explain, but stems mainly from differences in the way coding frames and orthology were defined. In Buschiazzo *et al.*[26] 100 comparisons revealed a dN/dS ratio higher than one, and most of these estimates are based on short alignments where both the number of synonymous sites and changes were small. This is in stark contrast to our results with very few values larger than one and a weaker correlation between dN/dS ratios and alignment length (Figure 5, Additional file 3). Buschiazzo *et al.*[26] also estimated dN/dS between *Arabidopsis thaliana* and *Populus trichocarpa* and obtained a value of 0.0924. They therefore concluded that dN/dS was higher in conifers than in angiosperms. While our results for the conifer data are within the same order of magnitude, it is worth noting that the estimate they obtained between *A. thaliana* and *P. trichocarpa* tends to be much lower than previous estimates from angiosperms. For example, in estimates comparing *A. thaliana* and *A. lyrata* the mean over more than 5000 genes was 0.203 and within the poplar lineage *P. trichocarpa* and *P. tremula* the mean over almost 600 genes was 0.175 [54,55]. It is also worth noting that the observed synonymous divergence between pine and spruce are more similar to the values obtained within the *Arabidopsis* and *Populus* lineages and since synonymous divergence seem to have a strong impact in estimates of dN/dS [56] comparing ratios over different groups of species should only be done when the observed synonymous divergence is similar. The latter, together with other uncertainty factors discussed above, suggests that caution is warranted before concluding that there are biologically meaningful differences in selective constraints between angiosperms and gymnosperms.

Even when the correct orthologous sequences have been aligned, individual estimates of dN/dS values should not be directly used to infer positive selection as recent work have highlighted several problems in using dN/dS larger than one as indicative of positive selection [56-58]. For example, the vast majority of human genes with dN/dS ratio larger than one is due to low dS values rather than high dN values. Hence, the classic interpretation of high dN/dS ratios is questionable as relatively low dS values will have the same effect on the ratio as high dN values. Furthermore, species pairs that are too closely related will tend to overestimate dN/dS.

In model organisms large scale studies of gene expression have identified numerous properties of genes and proteins that significantly correlate with selective constraints. Here we investigated whether level of gene expression in needles correlated with selective constraints, but found no significant pattern. In animals, fungi and plants a correlation between expression and selective constraint, such that highly expressed genes tend to have lower dN/dS, has been reported [54,59,60]. In multicellular organisms the correlation between dN/dS and expression breadth (number of tissues where genes are expressed) is often stronger than with the actual level of gene expression [54,55,61]. The lack of correlation in spruce could hence be due to the fact that we only measured gene expression in needles and have no data on expression breadth. Also, since our transcriptome reference sequence is largely created from short reads from needle RNA our reference is biased and contains a specific set of genes that do not fully represent the complete transcriptome; e.g our approach has limited information from genes lowly expressed in needles. The lack of correlation reported here should therefore be taken with a grain of salt as it might simply stem from lack of data rather than from an actual lack of correlation. Analysis of level of gene expression in samples collected in the dark compared to samples collected in the light revealed only a small set of genes showing at least a 2-fold difference in gene expression. Angiosperms studies suggest that around 20% of the transcriptome is differentially expressed between light and dark treatments, even though the number of genes varies depending on species, tissue and actual treatment [62-64]. The pattern observed in our data is different and might suggest that the diurnally expressed genes in gymnosperm trees could be fewer than in angiosperms. This is consistent with earlier studies, reporting a lack of clear diurnal expression pattern at key photosynthesis genes in gymnosperm species [65,66]. To be noted here is that all expression estimates put forward here stems from a single individual and is hence not suitable for making strong statements at the species level.

## Conclusions

Our study highlights the versatility of next generation sequencing technology in generation of full-length expressed genes, identification of polymorphic sites and estimation of gene expression levels. The available data from Norway spruce have with this study been leveraged with available data from North American spruce species. This allowed the comparison of sequence evolution in this group of plants for thousands of full-length genes. By comparing our *P. abies* assembly to other gymnosperm species our analysis suggests that evolutionary constraints might not, as some previous work have suggested, be very dissimilar between gymnosperm and angiosperm species. More importantly, our analysis does, together with the analysis of Buschiazzo *et al.*[26], highlights some of the problems associated with inference of selection from dN/dS in systems without annotated genome data. Even with a reference genome it is often difficult to unambiguously identify correct ORFs and orthologous sequences and one should consequently refrain from strong statements about patterns of divergence and selection in systems without high quality annotation data. However, as the collection of transcriptome sequence data is getting more streamlined and standardized (for example [67]) it will soon be possible to sample both more individuals and species and thereby facilitates more accurate characterization of selective constraints. The addition of more species will also make it feasible to pinpoint on which branches of evolutionary trees selection has been acting [5]. Furthermore, the recent in-depth analysis of factors contributing to protein evolution in model plants [54,61] will be within reach even in species without a reference genome and will hopefully reveal if there indeed is a difference in selective constraints between angiosperms and gymnosperms.

## Methods

### Plant material and RNA extraction

Needles from a single adult individual of Norway spruce growing naturally outside Uppsala, Sweden were collected at two different time points, at 1 pm during day light conditions and at 1 am the following night in the dark (20090527). The collected needles were actively growing and at stage 5 to 6 according to Krutzsch scale [68]. Extraction of total RNA was done from three replicates for each time point using 2.0 g of frozen needle tissue with the method described by Kolosova [69]. For all six samples around 400 *μ*g of total RNA was obtained of which close to 1% were retained as mRNA after two rounds of oligo-dT selection using the MicroPoly(A)Purist^*TM*^ (AppliedBiosystems).

### Preparation of cDNA libraries for cluster sequencing

A high concentration of random hexamer in relation to mRNA was used to synthesize cDNA with a short average fragment size using Invitrogen Supercript III First-Strand Synthesis kit. Following second strand synthesis, end-repairing of fragments, adaptors for short read pair-end sequencing supplied by Illumina corporation were attached to newly synthesized cDNA. The obtained cDNA library had an average size of 150-300 bp (the obtained length was actually shorter; see Results) and were used in ligation of adaptors for cluster sequencing. Enrichment of fragments with two attached adaptors was done using 2 *μ*l of purified ligation reaction in a 50 *μ*l PCR for 18 cycles. A final purification of the fragment libraries was done by separating the samples on agarose and performing a gel extraction with GE health cares Illustra kit. Before sequencing, the fragment sizes and concentrations were evaluated using Bioanalyzer 2100 and equimolar quantities of the library were used for sequencing on Illumina cluster station. A detailed description of the library preparation method can be found in [70].

### Sequence filtering and bioinformatic analysis

The raw sequence data were obtained using the Illumina python pipeline v. 1.3. The obtained sequences were then compared to a number of different potential sources of contamination, including the human transcriptome, rRNA data sets, chloroplast and mitochondrial genomes of spruce, and the genome of *E. coli*. In total, less than 5% of the reads mapped against these sequence libraries. Following this we created two sets of filtered sequence libraries, one for *de novo* assembly and one for mapping purposes. For both libraries we retained only high quality reads (quality > 35 for more than 90% of the read). For the *de novo* libraries we removed all reads showing traces of adaptor sequences, whereas for the mapping libraries we trimmed reads showing similarity to adaptor sequences and retained the read if it was longer than 30 bp. For the *de novo* library we also filtered artifact reads and removed any read with homopolymeric regions longer than 20 bp. We further removed the first 10 and last 11 bp of the reads, since the start of reads showed a different nucleotide composition compared to the rest of the sequence and the end of reads showed a decrease in quality (data not shown). This led to a *de novo* assembly with 33,185,901 reads of 55 bp length (∼15 million sequenced in both ends and ∼18 million from only one end), whereas mapping was done with 55,417,522 fragments (almost 36 millions sequenced in both ends and 19.5 million from only one end) of length between 30 and 76 bp.

Two different types of analyses of the read data were performed. First, *de novo* assembly of the obtained short read data was performed using a combination of two different assemblers followed by a step where putative unique contigs (PUTs) were created. We used two different programs and approaches for the *de novo* assembly since different assemblers often outcompete each other at different tasks [71]. The pair-end and single-end reads from all libraries were assembled using the short read assembler Velvet [35] using a combination of different k-mer lengths and expected coverages. In parallel, individual libraries were assembled as single end reads, treating forward and reverse reads as independent, using the program ABySS [36]. In total, this yielded one assembly from Velvet and 12 assemblies from ABySS. These assemblies and available EST data from Norway spruce were then merged into putative unique transcripts (PUTs) with the TGICL pipeline [72] if the identity of sequences were 95% or higher and showed at least 40 bp overlap. Second, we mapped the original short reads to the newly created PUTs which was used as a reference. Mapping was done with BWA [73] aligning pairs of reads and allowing maximum insert size to 500 bp and keeping only reads with mapping quality higher than 20.

In order to identify differentially expressed genes we used the BaySeq package [74], which is a part of the Bioconductor program suite. BaySeq implements an empirical Bayesian approach to estimate posterior probabilities of gene expression between treatments. The analysis was made on count data obtained by mapping our short read library to the created *P. abies* PUTs.

Finally, single nucleotide polymorphisms were detected using the mpileup function in SAMtools [75] on the short read alignment file against *P. abies* created with BWA. Since there is very limited knowledge about allele specific expression patterns we chose to only present SNPs with very high quality and having a estimated minor allele frequency in our short reads of at least 0.25.

All raw sequences is available at the NCBI SRA at the accession number SRA053572. A fasta file with with all PUTs as well as SNP information and PAML tables are available at DRYAD under DOI 10.5061/dryad.ds2gp.

### Analysis of selective constraints

The transcriptome sequence data used for inference of phylogenetic relationship and calculation of synonymous and non-synonymous divergence were collected from the following sources: EST sequences available at NCBI Genbank, PUTs assembled at http://plantgdb.org[37], our own sequence assembly and a short read assembly of *Taxus mairei*[19]. Sequences from *P. sitchensis* were downloaded from Genbank with the key word ’FLI’ and merged with Putative Unique Transcripts (PUTs) from plantgdb. *P. glauca* sequences came from the 27,720 transcripts from the *P. glauca* gene catalogue [22] and merged with PUT sequences from plantgdb. Sequences of *Pinus taeda* consisted of PUTs from plantgdb. The phylogenetic relationship between species was inferred using the program BEST [76] largely following the procedure described in [33], but running two chains for 10 million steps.

We used the program getorf from the Emboss software suite to get all possible ORF predictions falling between two stop codons (hereafter: stop1 for 5’ stop codon and stop2 for 3’ stop codon) [77]. Sequences were based on predictions assigned to six different categories: 1) presence of a start codon between stop1 and stop2; 2) only a start and stop2 codon present; 3) only a start and stop1 codon present; 4) only a stop2 codon present; 5) only a start and no stop codon present and finally 6) no start or stop codon present. Based on these categories we only considered category 1 as putative full-length ORFs and all others as partial ORFs (even though sequences assigned to category 2 could be full-length transcripts). We only kept ORFs after the start codon if stop2 was found and before stop1 if it was found. If the two longest ORF predictions on a given sequence had a length difference within 5 amino acids we kept the one assigned either as category 1 or 2 as the most likely ORF. For all other sequences we used the longest ORF in downstream analysis.

To evaluate our ORF prediction method, we tested our method on TAIR10 cDNA representative gene model (released on 2011-10-03) and compared our predictions to the peptide sequence database that consisted of 27,416 protein sequences. A total of 27,384 ORF predictions were found with good blastn quality against the protein database (median e-value = 0, median score = 717, median percentage of identity = 100% and both median query coverage and hit coverage = 100%). Among those, 26,867 ORFs have the best blast hit with their own proteins, which means that our procedure predicted the correct ORF in more than 98% of times.

Pairwise Reciprocal Blast hit approach was used to infer putative 1:1 orthologues between *Picea* species (*P. abies*, *P. glauca* and *P. sitchensis*) and *Pinus taeda* and *Taxus mairei* using blastn with threshold combination of e-value = 1×10^−05^, score = 200 and percentage of identity = 90% (for *Picea* vs *Pinus*) and 80% (for *Picea* vs *Taxus*). Peptides of predicted ORFs from both species were aligned using the program kalign [78]. To avoid alignment with too large divergence due to problematic ORF predictions from either species, we set a threshold for the percentage of identical amino acids to 50% and restricted the hits to contain less than 5 and 7 consecutive mismatches in *Picea* vs *Pinus* and *Picea* vs *Taxus*, respectively. This procedure resulted in a data set of putative orthologues between the different *Picea* species and either *Pinus taeda* or *Taxus* with all loci having E-values lower than 3 × 10^−50^ and 1 × 10^−50^, respectively. Nucleotide alignment was performed based on the protein alignments using revtrans [79]. Finally, dN/dS ratios between *Picea* and *Pinus*, and *Picea* and *Taxus* were estimated using codeml from PAML v. 4.4 [80], with settings seqtype = 1, runmode = -2, CodonFreq = 2 and transition-transversion ratio estimated from the data. Confidence intervals for the divergence estimates were obtained by bootstrap functions in R.

## Competing interests

The authors declare no competing interests.

## Authors’ contributions

TK, NG and UL designed the experiment. TK collected the needle material and did the wet-lab procedures. SU, JC and TK performed the bioinformatic analysis of the data. TK, JC, UL and ML drafted the manuscript. All authors contributed to and approved the final version of the manuscript.

## Supplementary Material

Additional file 1 Annotation file. Annotation and level of gene expression for the *Picea abies* PUTs. Details on the meaning of the different columns can be found on Sheet1. Click here for file

Additional file 2 Boxplot showing silent diversity estimates from different group of plant species. Click here for file

Additional file 3 ORF length and dN/dS. Comparison of the relationship between dN/dS and alignment length with Buschiazzo [26] data on the left and data from our study on the right. Click here for file
