# Supplementary figures and images for: Sequencing of the needle transcriptome from Norway spruce (Picea abies Karst L.) reveals lower substitution rates, but similar selective constraints in gymnosperms and angiosperms
Source: BMC Genomics. 2012 Nov 2;13:589. doi: 10.1186/1471-2164-13-589 (PMC3543189; doi:10.1186/1471-2164-13-589)

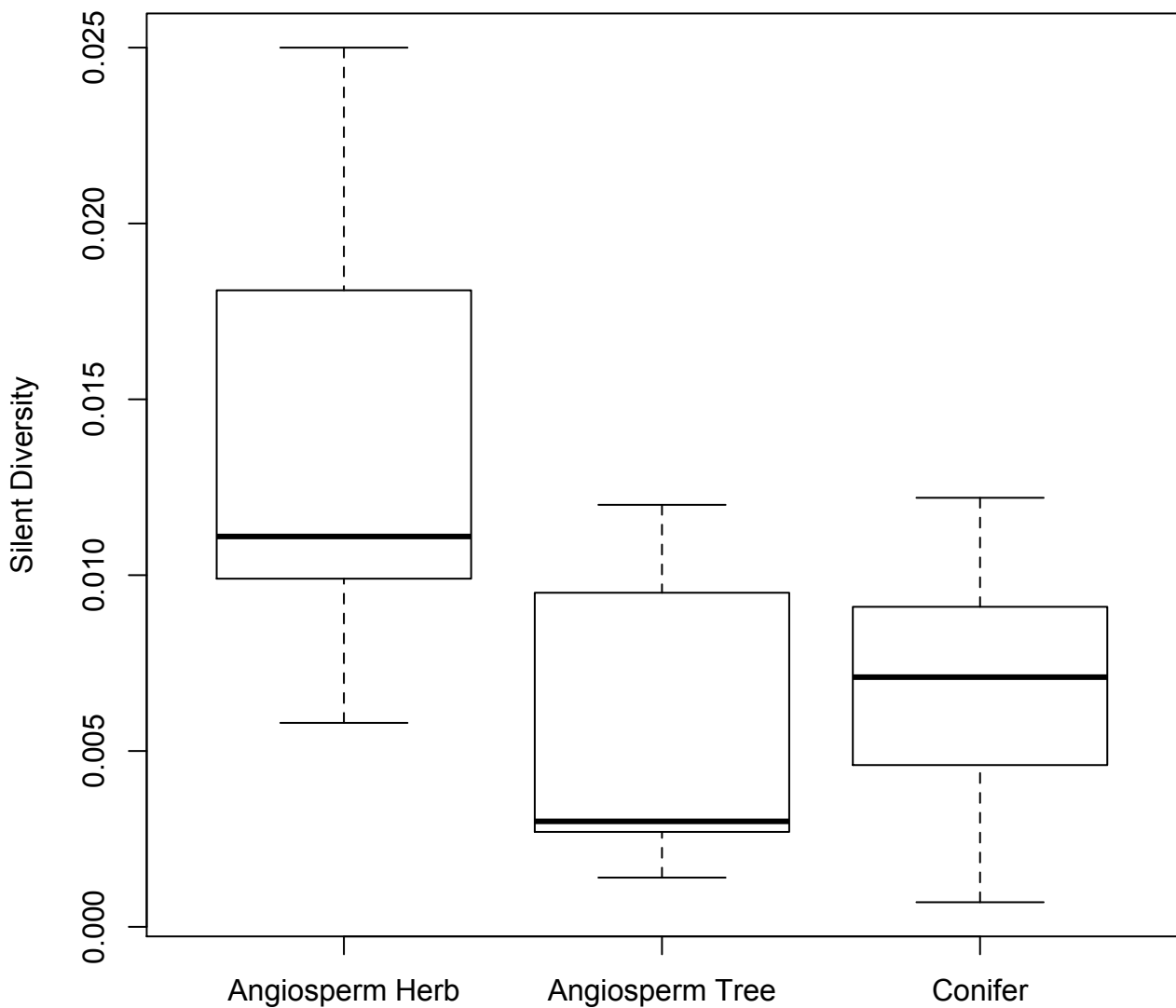

Supplement: Additional file 2 — Boxplot showing silent diversity estimates from different group of plant species. [file 1471-2164-13-589-S2.pdf]

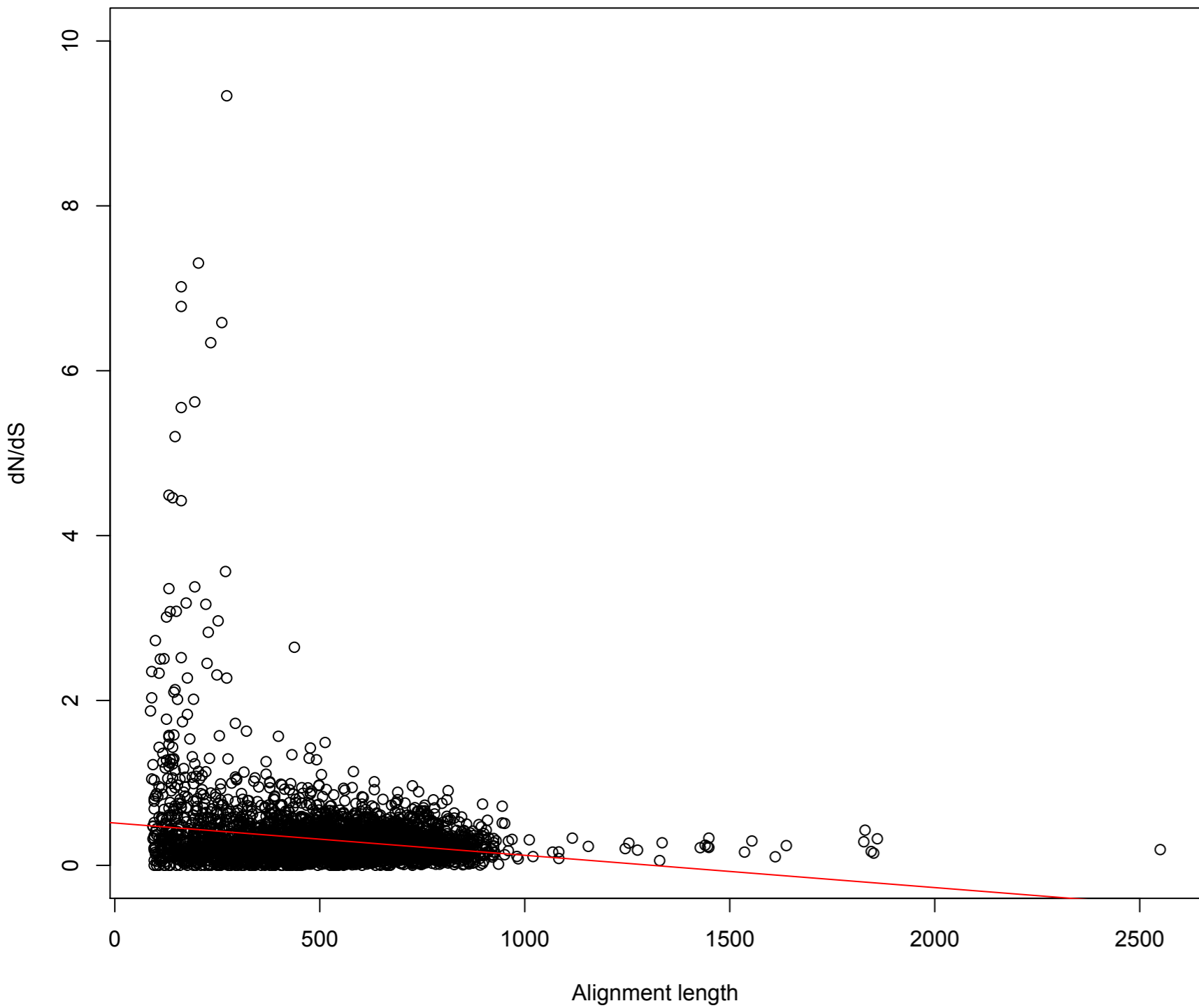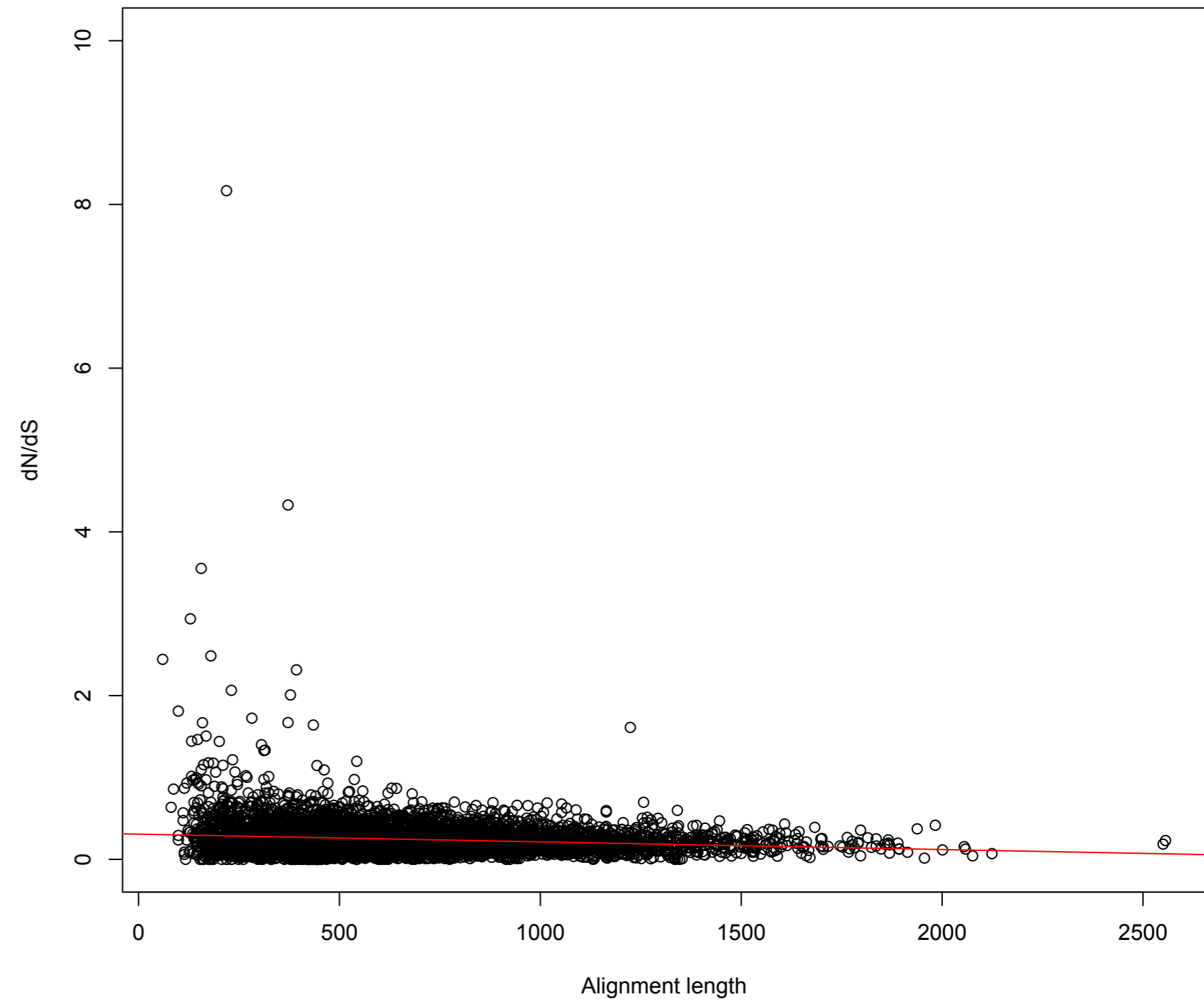

Supplement: Additional file 3 — ORF length and dN/dS. Comparison of the relationship between dN/dS and alignment length with Buschiazzo [26] data on the left and data from our study on the right. [file 1471-2164-13-589-S3.pdf]
